# Biofilm formation by designed co-cultures of *Caldicellulosiruptor* species as a means to improve hydrogen productivity

**DOI:** 10.1186/s13068-015-0201-7

**Published:** 2015-02-12

**Authors:** Sudhanshu S Pawar, Thitiwut Vongkumpeang, Carl Grey, Ed WJ van Niel

**Affiliations:** Division of Applied Microbiology, Lund University, Getingevägen 60, PO Box 124, SE-221 00 Lund, Sweden; Department of Biotechnology, Lund University, Getingevägen 60, PO Box 124, 221 00 Lund, Sweden

**Keywords:** Caldicellulosiruptor saccharolyticus, *Caldicellulosiruptor owensensi*s, Biohydrogen, Co-culture, c-di-GMP, UA reactor, CSTR, Volumetric H_2_ productivity

## Abstract

**Background:**

*Caldicellulosiruptor* species have gained a reputation as being among the best microorganisms to produce hydrogen (H_2_) due to possession of a combination of appropriate features. However, due to their low volumetric H_2_ productivities (*Q*_H2_), *Caldicellulosiruptor* species cannot be considered for any viable biohydrogen production process yet. In this study, we evaluate biofilm forming potential of pure and co-cultures of *Caldicellulosiruptor saccharolyticus* and *Caldicellulosiruptor owensensis* in continuously stirred tank reactors (CSTR) and up-flow anaerobic (UA) reactors. We also evaluate biofilms as a means to retain biomass in the reactor and its influence on *Q*_H2_. Moreover, we explore the factors influencing the formation of biofilm.

**Results:**

Co-cultures of *C. saccharolyticus* and *C. owensensis* form substantially more biofilm than formed by *C. owensensis* alone. Biofilms improved substrate conversion in both of the reactor systems, but improved the *Q*_H2_ only in the UA reactor. When grown in the presence of each other’s culture supernatant, both *C. saccharolyticus* and *C. owensensis* were positively influenced on their individual growth and H_2_ production. Unlike the CSTR, UA reactors allowed retention of *C. saccharolyticus* and *C. owensensis* when subjected to very high substrate loading rates. In the UA reactor, maximum *Q*_H2_ (approximately 20 mmol *·* L^−1^ *·* h^−1^) was obtained only with granular sludge as the carrier material. In the CSTR, stirring negatively affected biofilm formation. Whereas, a clear correlation was observed between elevated (>40 μM) intracellular levels of the secondary messenger bis-(3′-5′)-cyclic dimeric guanosine monophosphate (c-di-GMP) and biofilm formation.

**Conclusions:**

In co-cultures *C. saccharolyticus* fortified the trade of biofilm formation by *C. owensensis*, which was mediated by elevated levels of c-di-GMP in *C. owensensis*. These biofilms were effective in retaining biomass of both species in the reactor and improving *Q*_H2_ in a UA reactor using granular sludge as the carrier material. This concept forms a basis for further optimizing the *Q*_H2_ at laboratory scale and beyond.

**Electronic supplementary material:**

The online version of this article (doi:10.1186/s13068-015-0201-7) contains supplementary material, which is available to authorized users.

## Introduction

Amid the findings of vast reserves of shale oil and convenient negligence towards its (alleged) side-effects on the environment, the utopian world of ‘hydrogen economy’ still looks distant. One of the key bottlenecks is the unavailability of economical and eco-friendly ways of hydrogen production. Credible research is underway for developing sustainable processes producing hydrogen through electrolysis of water using wind and solar power [1]. However, more alternatives are needed to complement these technologies. In this respect, fermentative hydrogen (biohydrogen) production at a higher temperature, thermophilic biohydrogen production, using renewable biomass can be a viable option.

*Caldicellulosiruptor* species belong to a group of extremely thermophilic obligate anaerobes, which possess a natural ability to produce hydrogen from a wide range of mono-, di-, and oligo-saccharides and raw materials [2-6]. In addition to this, various other beneficial metabolic features enable the genus *Caldicellulosiruptor* as one of the best, yet not ideal, groups of bacteria with the natural ability to produce H_2_ [7]. Within this genus, *Caldicellulosiruptor saccharolyticus* and *Caldicellulosiruptor owensensis* are two of the best-studied species, both known to produce H_2_ near the theoretical maximum of 4 mol · mol^−1^ [8,9].

However, increasing *Q*_H2_ (volumetric H_2_ productivity; mmol · L^−1^ · h^−1^) is one of the major challenges in developing a cost-effective biohydrogen process with *Caldicellulosiruptor* species. The *Q*_H2_ depends on various factors such as cell density, extent of substrate conversion, and reactor configuration. The cell density can be increased by retaining more cells through different approaches, such as immobilization, cell entrapment, or cell retention. However immobilized or trapped cells can face mass transfer issues [10]. In contrast, biofilms, are well-organized structures, and are inherent to cell retention [11,12]. Moreover, biofilms generally follow ‘feed-and-bleed’ cycles allowing cell growth, which can be significant for growth-dependent product formation [11]. Among *Caldicellulosiruptor* species, *C. owensensis* has been previously reported to form biofilms [13] mainly by flocculating at the bottom of the reactor. However, no further information could be found regarding the factor(s) leading to biofilm formation by *C. owensensis* [13]. Bis-(3′-5′)-cyclic dimeric guanosine monophosphate (c-di-GMP) has been recognized as a ubiquitous secondary messenger in bacteria with multilayer control, i.e. at transcriptional, translational, and posttranslational level [14,15]. The c-di-GMP is synthesized using two molecules of guanosine-5′-triphosphate (GTP) by the enzyme diguanylate cyclase (DGC) and is hydrolyzed by the enzyme phosphodiesterase (PDE) [15]. Numerous studies have proven that high intracellular levels of c-di-GMP promote expression of extracellular-matrix related components needed for biofilm formation [14-16].

So far, most of the research pertaining to biohydrogen has been performed to investigate the physiological properties of H_2_-producing microbes. These studies have mainly been performed in continuously stirred tank reactors (CSTR). However, CSTR systems do not allow cell retention. Hence, it is of paramount importance to evaluate alternative reactor types that can help retain the biomass. Several different reactor types, such as packed bed reactor [13], membrane bioreactor [17], anaerobic sequencing blanket reactor [18], trickle bed reactor [19], and up-flow anaerobic (UA) reactor [20] aiding cell retention have been reported to produce H_2_ at higher rates. In fact, UA reactors are widely exploited for studies pertaining to biogas production. Their medium recirculation loop aids in achieving higher substrate conversion and also allows cells to adhere to the biofilms flocculated at the bottom of reactor. On the other hand, in case of the CSTR, using carriers has been reported to increase *Q*_H2_ by several folds [21].

In this study we aimed to evaluate the biofilm forming potential of *C. saccharolyticus* and *C. owensensis* in pure culture, and also evaluate whether *C. owensensis* through biofilm formation aids *C. saccharolyticus* when cultivated in co-cultures. Furthermore, we report the intracellular levels of c-di-GMP in both the organisms and its relationship with biofilm formation. We also evaluate the potential of UA reactors in improving *Q*_H2_ compared to CSTRs and whether carrier materials affect retaining the biomass and improving *Q*_H2_.

## Material and methods

### Microorganism and its maintenance

*C. saccharolyticus* DSM 8903 and *C. owensensis* DSM 13100 were purchased from the Deutsche Sammlung von Mikroorganismen (DSM) und Zellkulturen (Braunschweig, Germany). Routine subcultures and maintenance were conducted in 250 mL serum bottles containing 50 mL of a modified DSM 640 medium [22] unless stated otherwise. Anoxic solutions of glucose, cysteine · HCl, and magnesium sulphate were autoclaved (1.5 atm, at 120°C for 20 minutes) separately and added to the sterile medium at the required concentration. A 1,000× concentrated vitamins solution was prepared as described previously [8] and used in the growth medium at 1× concentration as a replacement for yeast extract. A 1,000× concentrated trace element solution was prepared as described previously [23].

### Fermentation setup and culture medium

To study the effect of any excretion of *C. saccharolyticus* on the growth of *C. owensensis* and vice versa, batch cultures of each were performed in biological duplicates and previously collected cell-free culture supernatant of one organism was added into the batch medium of another prior to inoculation. The volume of supernatant added in each respective case was equivalent to that of containing 1 g cell dry weight (CDW) of the respective organism.

To study the effect of different reactor systems on biofilm formation and cell retention, *C. saccharolyticus* and *C. owensensis* were cultivated independently (pure culture) or together (co-culture) in two different reactor systems: continuously stirred tank reactor (CSTR) and up-flow anaerobic (UA) reactor (Table 1). To allow for biofilm formation and/or cell retention, co-cultures of *C. saccharolyticus* and *C. owensensis* were performed in both the reactor systems with K1-carriers (Catalogue # K1, AnoxKaldnes AB, Lund, Sweden). K1-carrier is made of polyethylene in a tube-like structure (length, 7.2 mm; diameter, 9.1 mm with an internal cross and 18 external fins. In the case of the CSTR, co-cultures were performed with or without stirring, however, the pure cultures were only performed without stirring but with the K1-carriers (Table 1). In the case of the UA reactor, the co-cultures were performed with and without using the granular sludge as the packed bed, however, the pure cultures were performed only with granular sludge (Table 1).

**Table 1:** **Various cultivation conditions applied during this study**

**Name**	**Cultivation condition**
Case A	Co-culture in CSTR* without stirring with carriers
Case B	Co-culture in CSTR with stirring without carriers
Case C	*C. saccharolyticus* without stirring with carriers
Case D	*C. owensensis* without stirring with carriers
Case E	Co-culture in UA** reactor with sludge
Case F	*C. saccharolyticus* in UA reactor with sludge
Case G	*C. owensensis* in UA reactor with sludge
Case H	Co-culture in UA reactor without sludge with carriers
Case I	Co-culture in UA reactor without sludge without carriers

*CSTR, continuously stirred tank reactor; **UA, up-flow anaerobic.

All experiments were conducted in a jacketed, 3 L (CSTR) or 1 L (UA), equipped with an ADI 1025 Bio-Console and an ADI 1010 Bio-Controller (Applikon, Schiedam, The Netherlands) at a working volume of 1 L (CSTR) or 0.85 L (UA), either in batch or continuous mode. The water height in the UA reactor was maintained at approximately 20 cm. The pH was maintained at 6.5 ± 0.1 at 70°C by automatic titration with 4 M NaOH. The temperature was thermostatically kept at 70 ± 1°C. In case of the CSTR, a condenser with 5°C cooling water was fitted to the bioreactor’s headplate and the stirring was kept at 250 rpm unless specified otherwise. The UA reactor’s top was fitted with a rubber cork inserted with a collection tube releasing the flue gas out of the reactor. During batch cultivations, culture samples were collected at different time intervals for monitoring growth, and the culture supernatant was collected for analysis of glucose, acetic acid, lactic acid, propionic acid, and ethanol. Gas samples were collected from the headspace to analyze levels of H_2_ and CO_2_. During continuous cultures, samples for c-di-GMP were collected at steady state. Batch cultures were performed in two independent biological replicates, whereas, for continuous cultures steady states were obtained in technical duplicates.

All the reactors were autoclaved with a base medium (BM) containing per litre of demineralized water: KH_2_PO_4_ 0.75 g; K_2_HPO_4_ · 2H_2_O 1.5 g; NH_4_Cl 0.9 g; yeast extract 1.0 g; resazurin 1 mg; 1000 × modified SL-10 1 mL. Solutions of glucose, 10 g · L^−1^ for CSTRs (Case A, B, C, and D) and 20 g · L^−1^ for UA reactors (Case E. F, G, H, and I), cysteine · HCl, 0.25 g · L^−1,^ and MgSO_4_ · 6H_2_O, 0.5 g · L^−1^ were autoclaved and added separately prior to inoculation. UA reactors containing 250 g of granular sludge as a carrier material (Case E, F, and G) were autoclaved twice to eliminate the risk of methanogenic or hydrogenogenic contaminants. Autoclaving conditions did not affect the shape or the integrity of the granules. Gas samples were regularly taken from the headspace of UA reactors to detect any traces of methane. Carriers were autoclaved separately and were added prior to inoculation. The granular sludge was obtained from methanogenic reactors treating municipal waste water under mesophilic conditions. The granules of anaerobic sludge were circular in shape, measuring about 2 mm in diameter. Inocula for each organism were prepared through a succession of at least three sub-cultivations prior to inoculation. In the case of co-cultures, inocula of each organism were grown separately.

For continuous cultivations, the bioreactor started to be fed with fresh medium at the end of the logarithmic growth phase of the batch culture. Glucose was used as a primary substrate in all continuous experiments at an initial concentration of 10 g · L^−1^. Steady states were assessed after at least five volume changes based on the criteria of constant H_2_ and CO_2_ production rates and constant biomass concentration.

### Analytical methods

Headspace samples were analyzed for CO_2_, H_2_, and CH_4_ by gas chromatography, using a dual channel Micro-GC (CP-4900; Varian, Micro gas chromatography, Middelburg, The Netherlands), as previously described [8]. The results were analyzed with a Galaxie Chromatography Workstation (version 1.9.3.2, Middelburg, The Netherlands). The optical density of the culture was measured at 620 nm (OD_620_) using a U-1100 spectrophotometer (Hitachi, Tokyo, Japan). CDW was determined by filtration as previously described [24]. Glucose, acetate, lactate, propionate, and ethanol were analyzed by HPLC (Waters, Milford, Massachusetts, United States) on an Aminex HPX-87H ion exchange column (Bio-Rad, Hercules, United States) at 45°C, with 5 mM H_2_SO_4_ (0.6 ml · min^−1^) as the mobile phase. The column was equipped with a refractive index detector (RID-6A; Shimadzu, Kyoto, Japan).

### Scanning electron microscopy of biofilm samples

Biofilm samples were scraped from the pH probe and/or carrier at the end of the cultivation (Case A) and were immediately stored overnight in glutaraldehyde solution (2 to 3%) to allow fixation. The samples were then stored with sodium cacodylate buffer (about pH 7) until further use. A few hours prior to scanning electron microscopy (SEM) imaging, samples were dried by first washing with ethanol solutions from 50 to 100% in series and then subjecting to ‘critical point drying’ using liquid CO_2_. Subsequently, the dry biofilm samples were then glued on a stub and were sputter coated with gold/palladium alloy and finally viewed under the SEM (Hitachi SU3500, Hitachi, Japan).

### Determination of intracellular levels of c-di-GMP

During batch cultivations, 5 mL of culture samples were collected in quadruplets when cultures reached stationary phase. Similarly, in continuous cultures, 5 mL of culture samples were collected in quadruplets at steady state obtained under various conditions. All samples were collected on ice and were centrifuged immediately at 4000 rpm in a swinging bucket rotor at 4°C and were subsequently processed for the extraction of c-di-GMP. The extraction was performed as described by [25] with the exception that in the final step, samples were dried by incubating overnight at approximately 50°C.

The quantification of c-di-GMP was performed as previously described [25] but with the following modifications. The LC-separation were performed using isocratic conditions, 3.5% MeOH (A) and 96.5% 10 mM ammonium acetate in 0.1% acetic acid (B) at 400 μL/min for 6.5 min. The internal standard, xanthosine 5'- monophosphate (XMP), eluted after 3.1 min and c-di-GMP at 4.7 min. A wash program was run every 16 samples to ensure a robust analysis, in which 90% A was applied for 15 min before equilibrating the column for 20 min using the isocratic conditions. Standards, seven levels, ranging from 10 nM to 10 μM were included in the beginning and end of the sequence. The detection was performed using an Orbitrap-Velos Pro mass spectrometer (Thermo Scientific, Waltham, MA, USA) using the electrospray ionization (ESI) in positive mode. Two scan events were applied: ion trap (ITMS) for quantification, including selected reaction monitoring (SRM) on XMP (m/z 347/153 between 0 and 4 min) and c-di-GMP (m/z 691/540 between 4 and 6.5 min) and orbitrap fullscan (FTMS) for accurate mass identification, using a resolution of 30000.

### Bioinformatics analysis for genes related to bis-(3′-5′)-cyclic dimeric guanosine monophosphate

Genomes of *C. saccharolyticus* and *C. owensensis* were analyzed to locate genes coding for DGC and PDE. All the information regarding genome sequences and corresponding annotations were retrieved from the Integrated Microbial Genomes (IMG, Berkeley, United States).

### Population dynamics in the biofilm samples of co-cultures using qPCR

During all the co-culture experiments, 2 mL of culture samples were collected and immediately centrifuged and the cell pellets were stored at −20°C until further use. Similarly, sufficient amounts of biofilm samples were collected from the pH probe and from the reactor wall after the cultivations were ceased. The genomic DNA from the samples were extracted using Invitrogen’s (Carlsbad, United States) EasyDNA genomic DNA extraction kit (Catalogue number K1800-01) as per manufacturer’s protocol and stored at −20°C until further use.

To determine the relative presence of *C. saccharolyticus* and *C. owensensis* in the co-cultures, quantitative PCR (qPCR) assays were performed as described below. The 16S rDNA sequence was used as target for identification and quantification of each species. To design specific primers (Table 2), dissimilar regions were identified between target sequences using various sequence alignment tools available in the computer software BioEdit (Ibis Biosciences, Carlsbad, California, United States, 92008). PCR amplification and detection were performed in a LightCycler® Nano instrument (Roche Diagnostics, Mannheim, Germany). The PCR assay mixture (20 μL) contained: 1 × ExTaq buffer, 1U TaKaRa ExTaq HS DNA polymerase, 4.5 mM MgCl_2_, 0.2 mM dNTP (all from Th. Geyer GmbH, Renningen, Germany), 2 μg BSA, 1 × Eva green solution (Bioline GmbH, Luckenwalde, Germany), forward and reverse primers (each 0.5 μM, Table 2) and 4 μL of DNA template. For *C. saccharolyticus* the qPCR amplification protocol started with an initial denaturation at 95°C for 180 seconds, followed by 45 cycles of denaturation at 95°C for 10 seconds, annealing at 67°C for 10 seconds, and elongation and fluorescence acquisition at 72°C for 25 seconds. To confirm the absence of unspecific products, melting-curve analysis was performed as follows: heating at 60°C for 60 seconds followed by an increase in temperature by 0.1°C/s up to 97°C. Similar assays were performed for *C. owensensis*; albeit by changing the annealing temperature to 60°C. Quantification was performed using the method of absolute quantification with the help of LightCycler Nano software version 1.1. Pure genomic DNA samples (2.4 to 48 ng/μL) of each species were used in each run of the LightCycler Nano to establish a standard curve. Each run consisted of a blank assay with a PCR mixture containing dH2O instead of DNA template. It also consisted of a negative control assay with a PCR mixture containing the primers designed for one of the organisms from the pair of *Caldicellulosiruptor* species used in this study and genomic DNA of the other as a template and vice versa. For a particular sample, the DNA concentration of each species was added together and then their relative fractions were determined.

**Table 2:** **Primers used in this study**

**Organism (Locus tag)**	**Primer**	**Sequence**	**Product (bp)**
*C. saccharolyticus* (Csac_R0001)*	F_R0001	GGTGCGTAGGCGGCTATGCG	448
	R_R0001	CCCACCCTTTCGGGCAGGTC	
*C. owensensis* (Calow_R0003)	F_R0003	GCTAAGCGGATGGGGGAAACT	582
	R_R0003	CTGGCAGTGTTGAACGC	

*Primers for *C. saccharolyticus* were obtained from a previous study [31].

### Calculations

The *Q*_H2_ (mmol · L^−1^ · h^−1^) and cumulative H_2_ formation (CHF, mmol · L^−1^) were calculated in two different ways, depending on the experimental design. All calculations were based on the ideal gas law and the H_2_ and CO_2_ concentrations in the headspace. For the cultures in the CSTR, the calculations were based on the flow rate of the influent N_2_ gas and the percentages of H_2_ and CO_2_ in the effluent gas, as no other gases were detected. Thus, *Q*_H2_ and CHF were calculated based on hydrogen concentration in the effluent gas and the flow rate of the effluent gas. For the experiments performed in the UA reactor, the *Q*_H2_ was assumed to be twice the respective acetate productivities based on the stoichiometry [26]. Product yields were calculated by determining moles of products formed per mole of glucose consumed. Biomass yield was calculated as moles of biomass formed per mole of glucose consumed. Carbon and redox balances were calculated as described previously [9].

## Results

### Results obtained from continuously stirred tank reactors

**Figure 1:**
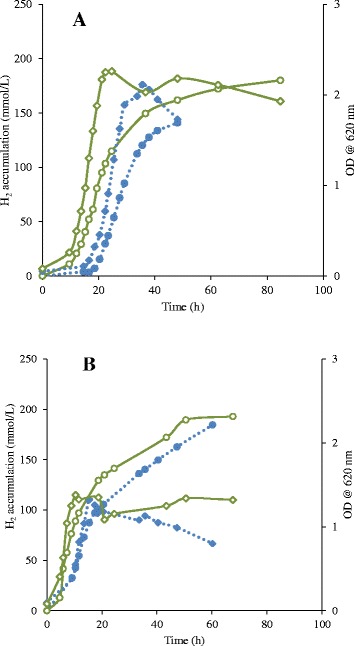
**Growth and H**_**2**_ **accumulation by** ***C. saccharolyticus*** **and** ***C. owensensis*** **in pH-controlled batch fermentations.** Presence (solid green line) and absence (dotted blue line) of each other’s supernatant, *C. saccharolyticus* **(A)** and *C. owensensis* **(B)**. Optical density (OD) measured at 620 nm when grown with supernatant (open diamond) and without supernatant (filled diamond); H_2_ accumulation when grown with supernatant (open circles) and without supernatant (filled circles).

Pure cultures in batch mode were tested for the influence of excretory metabolites from one species to another. For this reason, the supernatant of one organism was added to the reactor of the other prior to inoculation. As a control, both organisms were also grown in pure culture in absence of each other’s supernatant. Batch cultures of both *C. saccharolyticus* and *C. owensensis* displayed significantly shorter lag phases when grown in the presence of each other’s supernatant rather than in absence of it (Figure 1A and B). Moreover, when exposed to each other’s supernatant the cultures accumulated higher amounts of H_2_ and biomass, and were less prone to cell lysis in the stationary phase (Figure 1A and B). These are clear indications that both species might influence each other when in co-culture.

**Figure 2:**
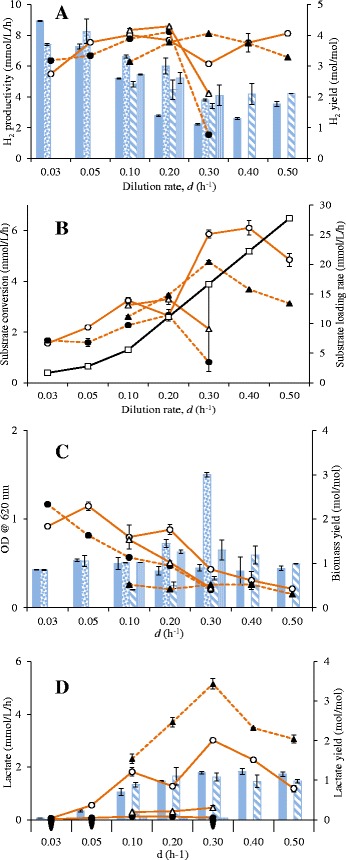
**Results of the continuous cultures of** ***C. saccharolyticus*** **and** ***C. owensensis*** **performed in the continuously stirred tank reactor (CSTR). (A)** *Q*_H2_, line graph (mmol · L^−1^ · h^−1^) and H_2_ yield, bar graph (mol · mol^−1^)_;_ **(B)** substrate conversion rate (mmol · L^−1^ · h^−1^); **(C)** Optical density (OD) measured at 620 nm, line graph, and biomass yield, bar graph (mol/mol) from planktonic phase; and **(D)** lactate productivity (mmol · L^−1^ · h^−1^), line graph, and lactate yield (mol · mol^−1^), bar graph. Case A (open circles, filled bar); Case B (filled circles, dotted bar); Case C (open triangles, bar with vertical lines); and Case D (filled triangles, bar with skewed lines). Substrate loading rate, solid black line with open squares.

To evaluate the biofilm-forming potential and its effect on biomass retention, *Q*_H2_, substrate conversion rate, and lactate formation by *C. saccharolyticus* and *C. owensensis*, experiments were performed in the CSTR with or without K1-carriers (Cases A to D, Table 1). In continuous cultures performed in the CSTR, maximum *Q*_H2_ and maximum substrate conversion were obtained in Case A, whereas, maximum lactate productivity was observed in Case D (Figure 2A, B and D). Cultures of Case A and D sustained growth at higher dilution rate, d (h^−1^), than those of Case B and C (Figure 2C). In case of *Q*_H2_, no particular trend was observed for Case A with increasing d (h^−1^), whereas, for Case B and C the *Q*_H2_ increased until d = 0.2 h^−1^ and then decreased. For Case D, *Q*_H2_ increased until d = 0.3 h^−1^ and then slightly decreased. The hydrogen yield was at its theoretical maximum only at low d (0.03 to 0.05 h^−1^). Generally, for all the continuous cultures performed in the CSTR, the H_2_ yield decreased with increasing d (h^−1^), with the exception of Case A where it slightly increased at d >0.3 h^−1^ (Figure 2A). For Case A, the substrate conversion rate (SCR) increased with increasing substrate loading rate (SLR). For Cases B and C, the SCR increased with increasing SLR until d = 0.2 h^−1^ and then dropped. Similarly, for Case D, the SCR increased with increasing SLR until d = 0.3 h^−1^ and then decreased. For all the continuous cultures performed in the CSTR (Cases A to D) at 0.05 > d > 0.4 h^−1^, in most cases the SLR was always higher than the SCR (Figure 2B).

For all the continuous cultures performed in the CSTR, the planktonic biomass concentration generally decreased with increasing d (h^−1^) (Figure 2C). At any particular d (h^−1^), Case A generally accumulated more planktonic biomass than Cases B, C, or D. Considering the pure cultures, *C. saccharolyticus* (Case C) showed higher biomass concentration compared to *C. owensensis* (Case D). Surprisingly, for Case B, the biomass yield suddenly increased at 0.3 h^−1^, but was non-existent at higher d due to washout. No particular trend was observed in biomass yields with increasing d (h^−1^) for Case A, C, or D. The cultures of Cases A and D could not sustain growth at d >0.5 h^−1^, whereas, cultures of Case B and C washed out at d >0.3 h^−1^ (Figure 2C). Of the co-cultures, lactate production was only observed when the culture was not stirred (Case A), and increased with the d until 0.3 h^−1^ where it decreased thereafter. Similarly, for the pure cultures, only *C. owensensis* (Case D) produced significant amounts of lactate, which increased with the d until 0.3 h^−1^ and decreased thereafter. A similar trend was observed with the lactate yield for Cases A and D. Overall, the CSTR appeared to be an inappropriate system with respect to achieving higher SCR and *Q*_H2_. Therefore, another reactor type was used for further studies.

### Results obtained from continuous cultures in the up-flow anaerobic reactor

**Figure 3:**
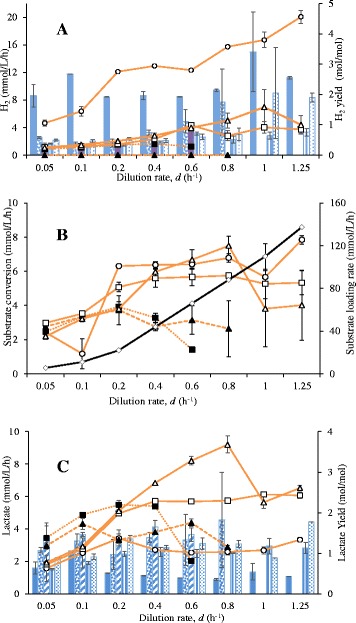
**Results of the continuous cultures of** ***C. saccharolyticus*** **and** ***C. owensensis*** **performed in the up-flow anaerobic (UA) reactors. (A)** *Q*_H2_, line graph (mmol · L^−1^ · h^−1^) and H_2_ yield, bar graph (mol · mol^−1^)_;_ **(B)** substrate conversion rate and substrate loading rate (mmol · L^−1^ · h^−1^); and **(C)** lactate productivity (mmol · L^−1^ · h^−1^), line graph and, lactate yield (mol · mol^−1^), bar graph. Case E (open circles, filled bar); Case F (open squares, open bar); Case G (open triangles, bar with vertical lines); Case H (filled triangles, dotted bar); and Case I (filled squares, bar with horizontal lines). Substrate loading rate, solid line with open squares.

Again, to evaluate the biofilm-forming potential and its effect on biomass retention, *Q*_H2_, substrate conversion rate, and lactate formation by *C. saccharolyticus* and *C. owensensis*, experiments were performed in a UA reactor with either granular sludge or K1-carriers as carrier materials (Cases E to H, Table 1), or without any carrier (Case I, Table 1). The highest *Q*_H2_ (approximately 20 mmol · L^−1^ · h^−1^) was obtained in a co-culture with granular sludge at a d = 1.25 h^−1^ (Case E, Figure 3A). The *Q*_H2_ of this culture increased steadily with increasing d (h^−1^) and was higher than any other culture performed in the UA reactor at any particular d (h^−1^). Other co-cultures, with and without K1-carriers, produced H_2_ at significantly lower rates, but without any particular trend with increasing d (h^−1^). On the other hand, the pure cultures of both organisms in the presence of granular sludge (Case F and G) produced H_2_ at higher rates than the co-cultures without granular sludge (Case H and I, Figure 3A). Among these pure cultures no significant differences were observed in *Q*_H2_ at any d (h^−1^) except at 0.8 and 1.0 h^−1^, where *C. owensensis* (Case G) displayed a slightly higher *Q*_H2_ (Figure 3A). The H_2_ yields were the highest for the co-culture with granular sludge compared to all other cultures at any particular d (h^−1^) and generally varied between 2 and 3.3 mol of H_2_/ mol of glucose consumed (Figure 3A). The SCR in the UA reactor with granular sludge (Case E, F, and G) generally increased with the SLR (at d ≤0.8 h^−1^) (Figure 3B). Even though cultures with granular sludge (Case E, F, and G) survived SLR values up to 140 mmol · L^−1^ · h^−1^, none of them displayed SCR more than 10 mmol · L^−1^ · h^−1^. At d >0.1 h^−1^, cultures without granular sludge (Cases H and I) could not sustain growth at SLR values beyond approximately 90 mmol · L^−1^ · h^−1^ and generally displayed much lower SCR compared to cultures with granular sludge (Case E, F and G, Figure 3B).

All liquid samples withdrawn from the granular sludge containing cultures (Case E, F, and G) contained sludge granules, which made it difficult to determine the planktonic biomass concentration, thus no reliable data could be obtained. On the other hand, planktonic biomass concentration in cultures without granular sludge was very low (data not shown), as is evident from the low SCR values obtained in these cultures (Case H and I, Figure 3B).

The highest lactate productivity was observed in the *C. owensensis* culture with granular sludge (Case G, Figure 3C). At d >0.2 h^−1^, both the pure cultures with granular sludge (Case F and G) displayed higher lactate productivity than the co-culture with (Case E) or without sludge (Case H and I). Of these co-cultures, the one without granular sludge (Case H and I) produced lactate at higher rates than the one with granular sludge (Case E). The lactate yields were lowest for the co-culture with granular sludge (Case E) at any particular d (h^−1^)*.* No significant differences in lactate yield were observed among the other cultures (Case F, G, H, and I).

### Biofilm formation by *Caldicellulosiruptor* species

**Figure 4:**
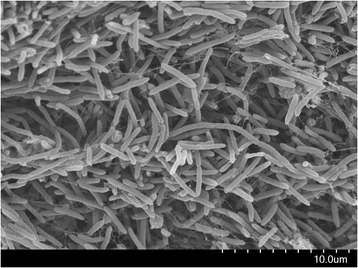
**SEM image of a biofilm obtained from the pH probe from the co-culture (Case A).**

No biofilm was observed during any of the batch cultures performed. In the continuous cultures, at d >0.2 h^−1^ a substantial amount of flocculation was observed at the bottom of the CSTR in the co-culture when stirring was not applied (Case A, Additional files 1 and 2). In addition, in this culture at d >0.2 h^−1^, biofilm was also observed on the reactor walls, pH probe, and K1-carriers. In contrast, when stirring was applied (Case B), no biofilm was observed. Among the pure cultures, no biofilm was observed on the reactor wall, pH probe, or K1-carriers in either of the Cases C and D. However, a biofilm in the form of flocculation of cells was observed in the *C. owensensis* culture for the entire duration (Case D). When viewed under SEM, the biofilm growing on the pH probe of the CSTR with co-culture (Case A) revealed distinct cells attached to each other with visible fibre-like structures (Figure 4). Two different kinds of cell structures were observed, one as rod-shaped and unicellular form with dimensions 0.2 to 0.4 μm by 3 to 4 μm, whereas the other in a chain-like, multi-cellular structure with similar width (0.2 to 0.4 μm) but variable length depending on the number of cells in a chain (Figure 4).

The co-culture with sludge (Case E) displayed significant flocculation and biofilm on the reactor wall which was especially pronounced at d >0.2 h^−1^. Among the pure cultures, the *C. owensensis* culture with sludge (Case G) also displayed significant flocculation atop the sludge bed but hardly any biofilm was observed on the reactor walls. The co-cultures without sludge also displayed traces of biofilm on the reactor wall (Case H and I), however, no significant biofilm was observed on the K1-carriers (Case H).

### Intracellular levels of bis-(3′-5′)-cyclic dimeric guanosine monophosphate

**Figure 5:**
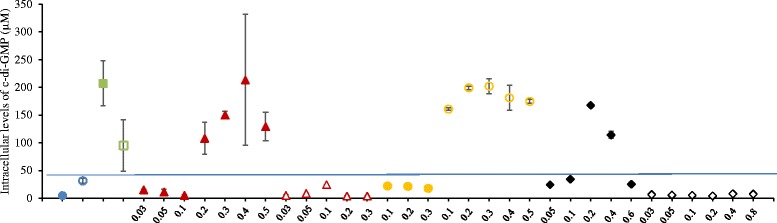
**Intracellular levels of c-di-GMP in batch and continuous cultures performed in CSTR and UA reactors.** Batch cultures without supernatant: *C. saccharolyticus* (filled circle, green), *C. owensensis* (filled square, green); batch cultures with each other’s supernatant: *C. saccharolyticus* (open circle, blue), *C. owensensis* (open square, green); Continuous cultures: Case A (filled triangle, red); Case B (open triangle, red); Case C (filled circle, yellow); Case D (open circle, yellow); Case I (filled diamond, black); and Case H (open diamond, black). For continuous cultures, the values on X-axis represent d (h^−1^) at which the sample was collected.

**Figure 6:**
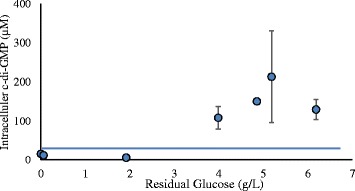
**Correlation between intracellular c-di-GMP levels and residual sugar concentration in the co-culture (Case A).**

The genomes of *C. saccharolyticus* and *C. owensensis* contain multiple genes coding for diguanylate cyclase (DGC), and phosphodiesterase (PDE) (Additional file 3). In batch cultures of *C. saccharolyticus* cells contained very low c-di-GMP levels compared to those observed in cells of *C. owensensis* (Figure 5). Interestingly, when grown in the presence of each other’s supernatant, cells of *C. saccharolyticus* accumulated higher levels of c-di-GMP compared to those cells grown without the supernatant of a *C. owensensis* culture (Figure 5). In contrast, the opposite trend was observed for *C. owensensis*. In continuous cultures, the co-culture without stirring (Case A) accumulated very low (<20 μM) levels of c-di-GMP at d ≤0.2 h^−1^. However, at d ≥0.2 h^−1^ the same culture accumulated at least 5 to 10-fold higher levels of c-di-GMP, albeit with no particular trend. Interestingly, in the co-culture without stirring (Case A), the levels of c-di-GMP appear to have increased when levels of residual sugar increased beyond 2 g · L^−1^ (Figure 6), without any particular pattern. In contrast, the co-culture with stirring accumulated very low (>30 μM) levels of c-di-GMP regardless of the d (h^−1^). Among the pure cultures, cells of *C. owensensis* (Case D) accumulated similar levels to those observed in the co-culture without stirring (Case A) at d ≥0.2 h^−1^, but approximately 10-fold higher levels than those observed in cells of *C. saccharolyticus* (Case C, Figure 5).

Among the UA cultures, the co-culture without K1-carriers (Case I), except for d 0.2 and 0.4 h^−1^, cells accumulated very low (<30 μM) c-di-GMP levels. The co-culture with K1-carriers (Case H) contained very low (<30 μM) c-di-GMP levels regardless of the d (h^−1^) (Figure 5). No samples were collected from cultures performed with sludge (Case E, F, and G) due to contaminations from granular sludge.

### Population dynamics in co-cultures of *C. saccharolyticus* and *C. owensensis*

**Figure 7:**
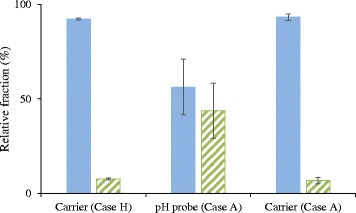
**Fraction of** ***C. saccharolyticus*** **and** ***C. owensensis*** **in biofilm samples (Case A and H).** *C. owensensis* (filled, blue) and *C. saccharolyticus* (horizontal lines, green), values on X-axis represent the source of the biofilm sample with respect to reactor system and the carrier.

In the co-culture without stirring performed in the CSTR (Case A), the biofilm on the pH probe consisted of *C. saccharolyticus* and *C. owensensis* in about a 1:1 ratio (Figure 7). However, in the same culture, the biofilm on the K1-carriers contained about 10 to 12 times more cells of *C. owensensis* than cells of *C. saccharolyticus*. Similarly, in the co-culture performed in the UA reactor (Case H), the biofilm on the K1-carriers contained the cell ratio of about 10:1 for *C. owensensis* compared to *C. saccharolyticus* (Figure 7). No results could be obtained with samples collected from planktonic cells in any of the cultures, possibly due to the low target DNA concentration.

## Discussion

### Effect of biofilm formation on Q_H2_, substrate conversion, and lactate formation

In a techno-economic analysis of a representative biohydrogen process, low *Q*_H2_ has been identified as a key bottleneck for making the process economically viable [27]. This study reports a higher *Q*_H2_ (approximately. 20 mmol · L^−1^ · h^−1^, Case E) than most of the previously obtained values in continuous cultures of *Caldicellulosiruptor* species [28], but which is still about an order of magnitude lower than the maximum *Q*_H2_ ever reported for thermophilic hydrogen producers [20]. Nevertheless, the highest maximum *Q*_H2_ in both these studies were obtained at very high d (>1.0 h^−1^), which may not be ideal for reasonable process economics [27]. Thus, further investigations are needed to determine the implications of high d (h^−1^) on a biohydrogen process.

Numerous studies have asserted that biofilm formation improves substrate conversion leading to increased *Q*_H2_ [20,21,29]. Similarly, in this study, formation of biofilm by co-cultures of *C. saccharolyticus* and *C. owensensis* improved the substrate conversion in the CSTR as well as the UA reactor (Case A and E). However, it had a varied effect on *Q*_H2_. In the UA reactor biofilm formation indeed improved *Q*_H2_. In the CSTR, however, improved substrate conversion was accompanied by an increase in lactate production (Case A), which consequently subdued *Q*_H2_. This abnormality of the CSTR accumulating relatively higher amounts of reduced by-products, such as lactate and ethanol, than UA reactors (Case A and E) was also observed in a similar study comparing conversion of wheat straw hydrolysate using mixed culture in CSTR and UA reactors [30]. In the present study, the aforementioned abnormality may have occurred due to the presence of a higher proportion of *C. owensensis* compared to *C. saccharolyticus* in the planktonic phase at high d (>0.2 h^−1^) in the CSTR. This hypothesis is supported by the fact that *C. owensensis* produced higher amounts of lactate than *C. saccharolyticus* regardless of the reactor system (Figure 2D and 3C), and that unlike the CSTR, the UA reactors inherently allow biomass retention, thus perhaps a higher fraction of cells of *C. saccharolyticus* were retained in the UA reactor compared to the CSTR when operated at higher d (h^−1^).

### Designed co-cultures versus pure cultures

Regardless of the reactor system used, the co-cultures converted higher amounts of substrate and, in the UA, displayed higher *Q*_H2_ than the pure culture of each species. This is in agreement with previous studies, where designed co-cultures of *C. saccharolyticus* and *Caldicellulosiruptor kristjanssonii* showed higher H_2_ yields than their pure cultures [31]. Similarly, a co-culture of *Clostridium thermocellum* JN4 and *Thermoanaerobacterium thermosaccharolyticum* GD17 reported two-fold higher *Q*_H2_ than either of their pure cultures [32], even though they are of different genus.

Both *Caldicellulosiruptor* species performed better in batch growth in the presence of each other’s supernatant, which clearly indicate that both species excrete compounds positively affecting the other one. A similar observation has been made for *C. saccharolyticus* excreting compound(s) that boosted the growth of *C. kristjanssonii* [31]. In fact, co-culture *C. saccharolyticus* boosted the growth performance of *C. kristjanssonii*, which can be interpreted as altruistic behaviour [31]. In the current study, a similar behaviour was seen with *C. saccharolyticus* fortifying *C. owensensis’* ability to form biofilm. On its turn, *C. owensensis* showed altruistic behaviour by aiding *C. saccharolyticus* to take part in the biofilm formation (Figure 6). This phenomenon is explained by ‘kin selection theory’ [33], according to which closely related species help each other to reproduce to pass its own genes on to next generation, even if indirectly. According to Hamilton’s rule, higher relatedness (r) between the species, higher fitness benefit (b) to the beneficiary, and lower fitness cost (c) to the altruist will ensure better cooperation (r × b – c >0) [33]. This may explain why the co-culture of *C. saccharolyticus* and *C. kristjanssonii* reported higher H_2_ yields [31] than any of the mixed cultures consisting of microorganisms of various genera ever reported. Indeed, another study argues simply that higher cooperation can be expected between highly related species [34].

Among the pure cultures, both *C. saccharolyticus* and *C. owensensis* produced higher amounts of lactate than previously reported studies [8,9] performed in similar conditions, except that stirring was not applied for the cultures in this study. Obviously, the non-stirring condition led to oversaturation of H_2_ and CO_2_ in the culture, leading to a shift in the metabolism [35,36]. Finally, the observation of an unusual increase in biomass yield in the pure culture of *C. saccharolyticus* (Case C) near its critical d (0.3 h^−1^) can be attributed to relatively higher energy spent by the culture on cell growth than product formation, as a reaction to wash-out conditions at a high d (h^−1^). A similar observation was reported in a previous study performed with *C. saccharolyticus* [23]. As far as we know, this has not been described before in the literature, and a clear rationale behind this phenomenon is lacking.

### Effect of reactor system and culture conditions

In UA reactors, only granular sludge provided a supporting bed to the flocculating biofilms of *C. owensensis* and *C. saccharolyticus*. This explains the very low *Q*_H2_ observed in the UA reactor without granular sludge. Similar results were obtained in a previous study performed with *Thermoanaerobacterium thermosaccharolyticum* PSU-2 [20]. However, despite its benefits, the risk of contamination from hydrogenotrophic methanogens threatens the stability of UA reactors when granular sludge is used. It could be that porous glass beads may be a viable alternative carrier. A recent study reported an increase in *Q*_H2_ and H_2_ yield by 70% and 30%, respectively, when cells of *Thermotoga neapolitana* were immobilized on porous glass beads in a CSTR [37].

**Figure 8:**
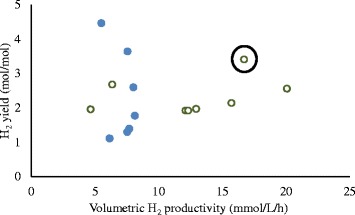
**The correlation between** ***Q***_**H2**_ **and H**_**2**_ **yield in co-cultures (Case A and E).** *Q*_H2_ (mmol · L^−1^ · h^−1^), H_2_ yield (mol · mol^−1^). Case A (filled circle, blue); Case E (open circle, red). The encircled data point represent the best case scenario where both *Q*_H2_ and H_2_ yield are reasonably high.

Although, higher *Q*_H2_ (>15 mmol · L^−1^ · h^−1^) is desirable for better process economics, a higher H_2_ yield (>3 mol · mol^−1^) can certainly contribute to improving the process economics when relatively expensive raw materials are used. In that respect, when the results obtained in this study are compared, UA reactors appear to offer a process alternative to achieve high *Q*_H2_ and yield (Figure 8). The CSTR, on the other hand, seems to have a boundary value around 10 mmol · L^−1^ · h^−1^ for *Q*_H2_ regardless of the H_2_ yield (Figure 8).

The UA reactor allowed d (h^−1^) well beyond the maximum specific growth rates of *C. saccharolyticus* and *C. owensensis* in pure and co-cultures, underlining the ability of UA reactors to retain the biomass of these species.

### Biofilm and intracellular levels of bis-(3′-5′)-cyclic dimeric guanosine monophosphate

A clear correlation was observed between the high intracellular c-di-GMP levels (>40 μM) and the stage of a particular culture initiating a biofilm. Although the samples were collected from planktonic biomass and not the biofilm itself, since the biofilms go through feed-and-bleed cycles, the planktonic cells can be assumed to be representative of the cells in the biofilm. Conversely, in the absence of any biofilm, very low c-di-GMP levels were observed when stirring was applied in continuous cultures in the CSTR (Case B). However, batch cultures of *C. owensensis* accumulated high levels of c-di-GMP but no biofilm was observed, perhaps due to the stirring. Moreover, c-di-GMP levels in co-culture performed without stirring (Case A) increased as the concentration of residual sugar increased beyond 2 g · L^−1^ (Figure 6). This may be because of a combination of the fact that the flocculating cells of *C. owensensis* at the bottom of the CSTR did not have access to the influent feed being dropped from the top of the CSTR, and that cells of *C. saccharolyticus* dominating the planktonic phase consumed most of the substrate until a d of 0.1 h^−1^, after which the residual concentration increased beyond 2 g · L^−1^ (Figure 6). Beyond that point the glucose gradient may have reached *C. owensensis* at the bottom, allowing the development of biofilms at d ≥0.2 h^−1^. Thus, it can be argued that if the co-cultures were performed at high substrate concentration, biofilm could have been obtained even at d <0.2 h^−1^. This knowledge may help in achieving SLRs as well as biofilms at low d (h^−1^), similar to those obtained at high d (h^−1^) in this study. However, the vulnerability of *C. saccharolyticus* to high osmotic pressure limits the option of performing cultures using feed with high substrate concentration [22]. Alternatively, a reactor system such as a UA reactor which feeds the influent from bottom may also be more appropriate, as shown in the present study.

Although, *C. saccharolyticus* possesses genes required for the synthesis of c-di-GMP, its intracellular levels are well below the critical level (40 μM). This perhaps explains the inability of *C. saccharolyticus* to form biofilm independent of *C. owensensis*. Arguably, overexpression of DGC may elevate the levels of c-di-GMP in *C. saccharolyticus*, allowing biofilm formation. Thus, encouraging *C. saccharolyticus* to form biofilms on its own may provide a better alternative to its co-culture with *C. owensensis*, considering the propensity of the latter to produce lactate and ethanol.

## Conclusions

Only when grown together in co-culture do, *C. saccharolyticus* and *C. owensensis* form substantial amounts of biofilm, improving substrate conversion and *Q*_H2_. Thus, such a constructed co-culture is an effective means to be exploited in any bioreactor designed for biomass retention, such as UA reactors. Indeed, UA reactors allow retention of *C. saccharolyticus* and *C. owensensis* when subjected to very high substrate loading rates, improving substrate conversion, and *Q*_H2_. Granular sludge showed superior support to biofilm formation in UA reactors. However, as sludge can be a potential source of methanogenic contaminants, it either needs proper pre-treatment, or more suitable alternatives should be found. Elevated intracellular levels of c-di-GMP are clearly linked to biofilm formation by *C. saccharolyticus* and *C. owensensis*. The maximum *Q*_H2_ obtained in this study was obtained at very high d (h^−1^) which may not be ideal for a reasonable process economics. Alternatively, a biofilm forming pure or co-cultures of *Caldicellulosiruptor* species, which can withstand feed containing high substrate concentrations, can be operated at a reasonably low d (h^−1^), which will allow similar substrate loading rates to that obtained in this study at high d (h^−1^). The way forward for industrial application is to further exploit the concept of this designed co-culture in UA-type reactors using granular sludge-type of carriers for obtaining higher volumetric hydrogen productivities.

## Additional files

Additional file 1:
**The planktonic biomass in the co-culture without stirring (Case A).** The boxes filled with different colours represent a particular d (h^−1^). Additional file 2:
**A short film showing the biofilm in action (Case A).**
Additional file 3: Table S1. Genes related to c-di-GMP synthesis and hydrolysis in *C. saccharolyticus* and *C. owensensis*.

## Abbreviations

c-di-GMP: bis-(3′-5′)-cyclic dimeric guanosine monophosphate
CDW: Cell dry weight
CHF: Cumulative H_2_ formation (mmol · L^-1^)
CSTR: Continuously stirred tank reactor
d: Dilution rate (h^-1^)
DSM: Deutsche Sammlung von Mikroorganismen
DGC: diguanylate cyclase
PDE: phosphodiesterase
XMP: xanthosine 5'- monophosphate
ESI: electrospray ionization
SRM: selected reaction monitoring
*Q*_*H2*_: Volumetric H_2_ productivity (mmol · L^-1^ · h^-1^)
SCR: Substrate conversion rate (mmol · L^-1^ · h^-1^)
SLR: Substrate loading rate (mmol · L^-1^ · h^-1^)
UA: Up-flow anaerobic

## References

[1] Delucchi MA, Jacobson MZ (2011). Providing all global energy with wind, water, and solar power, part II: reliability, system and transmission costs, and policies. Energy Policy..

[2] Pawar SS, Nkemka VN, Zeidan AA, Murto M, van Niel EWJ (2013). Biohydrogen production from wheat straw hydrolysate using *Caldicellulosiruptor saccharolyticus* followed by biogas production in a two-step uncoupled process. Int J Hydrog Energy..

[3] de Vrije T, Bakker RR, Budde MAW, Lai MH, Mars AE, Claassen PAM (2009). Efficient hydrogen production from the lignocellulosic energy crop Miscanthus by the extreme thermophilic bacteria *Caldicellulosiruptor saccharolyticus* and *Thermotoga neapolitana*. Biotechnol Biofuels..

[4] de Vrije T, Budde MAW, Lips SJ, Bakker RR, Mars AE, Claassen PAM (2010). Hydrogen production from carrot pulp by the extreme thermophiles *Caldicellulosiruptor saccharolyticus* and *Thermotoga neapolitana*. Int J Hydrog Energy..

[5] van de Werken HJG, Verhaart MRA, VanFossen AL, Willquist K, Lewis DL, Nichols JD (2008). Hydrogenomics of the extremely thermophilic bacterium *Caldicellulosiruptor saccharolyticus*. Appl Environ Microbiol..

[6] Ivanova G, Rákhely G, Kovács KL (2009). Thermophilic biohydrogen production from energy plants by *Caldicellulosiruptor saccharolyticus* and comparison with related studies. Int J Hydrog Energy..

[7] Pawar SS, van Niel EWJ (2013). Thermophilic biohydrogen production: how far are we?. Appl Microbiol Biotechnol..

[8] Zeidan AA, van Niel EWJ (2010). A quantitative analysis of hydrogen production efficiency of the extreme thermophile *Caldicellulosiruptor owensensis* OLT. Int J Hydrogen Energy..

[9] de Vrije T, Mars AE, Budde MAW, Lai MH, Dijkema C, de Waard P (2007). Glycolytic pathway and hydrogen yield studies of the extreme thermophile *Caldicellulosiruptor saccharolyticus*. Appl Microbiol Biotechnol..

[10] Kumar N, Das D (2001). Continuous hydrogen production by immobilized *Enterobacter cloacae* IIT-BT 08 using lignocellulosic materials as solid matrices. Enzyme Microb Technol..

[11] Karatan E, Watnick P (2009). Signals, regulatory networks, and materials that build and break bacterial biofilms. Microbiol Mol Biol Rev..

[12] Dufour D, Leung V, Lévesque CM (2010). Bacterial biofilm: structure, function, and antimicrobial resistance. Endod Topics..

[13] Peintner C, Zeidan AA, Schnitzhofer W (2010). Bioreactor systems for thermophilic fermentative hydrogen production: evaluation and comparison of appropriate systems. J Cleaner Prod..

[14] Hengge R (2009). Principles of c-di-GMP signalling in bacteria. Nat Rev Microbiol..

[15] Jenal U, Malone J (2006). Mechanisms of Cyclic-di-GMP Signaling in bacteria. Annu Rev Genet..

[16] Pérez-Mendoza D, Coulthurst SJ, Sanjuán J, Salmond GPC (2011). N-Acetylglucosamine-dependent biofilm formation in *Pectobacterium atrosepticum* is cryptic and activated by elevated c-di-GMP levels. Microbiology..

[17] Kim M-S, Lee D-Y, Kim D-H (2011). Continuous hydrogen production from tofu processing waste using anaerobic mixed microflora under thermophilic conditions. Int J Hydrog Energy..

[18] Prasertsan P, O-Thong S, Birkeland N-K (2009). Optimization and microbial community analysis for production of biohydrogen from palm oil mill effluent by thermophilic fermentative process. Int J Hydrog Energy..

[19] van Groenestijn JW, Geelhoed JS, Goorissen HP, Meesters KPM, Stams AJM, Claassen PAM (2009). Performance and population analysis of a non-sterile trickle bed reactor inoculated with *Caldicellulosiruptor saccharolyticus*, a thermophilic hydrogen producer. Biotechnol Bioeng..

[20] O-Thong S, Prasertsan P, Karakashev D, Angelidaki I (2008). High-rate continuous hydrogen production by *Thermoanaerobacterium thermosaccharolyticum* PSU-2 immobilized on heat-pretreated methanogenic granules. Int J Hydrog Energy..

[21] Koskinen PEP, Lay C-H, Puhakka JA, Lin P-J, Wu S-Y, Örlygsson J (2008). High-efficiency hydrogen production by an anaerobic, thermophilic enrichment culture from an Icelandic hot spring. Biotechnol Bioeng..

[22] Willquist K, Claassen PAM, van Niel EWJ (2009). Evaluation of the influence of CO_2_ on hydrogen production in *Caldicellulosiruptor saccharolyticus*. Int J Hydrog Energy..

[23] Pawar SS, Van Niel EWJ (2014). Evaluation of assimilatory sulphur metabolism in *Caldicellulosiruptor saccharolyticus*. Bioresour Technol..

[24] Willquist K, van Niel EWJ (2010). Lactate formation in *Caldicellulosiruptor saccharolyticus* is regulated by the energy carriers pyrophosphate and ATP. Metab Eng..

[25] Spangler C, Böhm A, Jenal U, Seifert R, Kaever V (2010). A liquid chromatography-coupled tandem mass spectrometry method for quantitation of cyclic di-guanosine monophosphate. J Microbiol Methods..

[26] Shen N, Zhang F, Song X-N, Wang Y-S, Zeng RJ (2013). Why is the ratio of H_2_/acetate over 2 in glucose fermentation by *Caldicellulosiruptor saccharolyticus*?. Int J Hydrog Energy..

[27] Ljunggren M, Wallberg O, Zacchi G (2011). Techno-economic comparison of a biological hydrogen process and a 2nd generation ethanol process using barley straw as feedstock. Bioresour Technol..

[28] Bielen AAM, Verhaart MRA, van der Oost J, Kengen SVM (2013). Biohydrogen production by the thermophilic bacterium *Caldicellulosiruptor saccharolyticus*: current status and perspectives. Life..

[29] Kim JO, Kim YH, Ryu JY, Song BK, Kim IH, Yeom SH (2005). Immobilization methods for continuous hydrogen gas production biofilm formation versus granulation. Process Biochem..

[30] Kongjan P, Angelidaki I (2010). Extreme thermophilic biohydrogen production from wheat straw hydrolysate using mixed culture fermentation: effect of reactor configuration. Bioresour Technol..

[31] Zeidan A, Rådström P, van Niel E (2010). Stable coexistence of two *Caldicellulosiruptor* species in a de novo constructed hydrogen-producing co-culture. Microb Cell Fact..

[32] Liu Y, Yu P, Song X, Qu Y (2008). Hydrogen production from cellulose by co-culture of *Clostridium thermocellum* JN4 and *Thermoanaerobacterium thermosaccharolyticum* GD17. Int J Hydrog Energy..

[33] Hamilton WD (1964). The genetical evolution of social behaviour. I. J Theor Biol..

[34] West SA, Griffin AS, Gardner A, Diggle SP (2006). Social evolution theory for microorganisms. Nat Rev Microiol..

[35] van Niel EWJ, Claassen PAM, Stams AJM (2003). Substrate and product inhibition of hydrogen production by the extreme thermophile, *Caldicellulosiruptor saccharolyticus*. Biotechnol Bioeng..

[36] Willquist K, Pawar SS, van Niel EWJ (2011). Reassessment of hydrogen tolerance in *Caldicellulosiruptor saccharolyticus*. Microb Cell Fact..

[37] Ngo TA, Bui HTV (2013). Biohydrogen production using immobilized cells of hyperthermophilic eubacterium *Thermotoga neapolitana* on porous glass beads. J Technol Innov Renewable Energy..

