# Supplementary material for: Biofilm formation by designed co-cultures of Caldicellulosiruptor species as a means to improve hydrogen productivity
Source: Biotechnol Biofuels. 2015 Feb 12;8:19. doi: 10.1186/s13068-015-0201-7 (PMC4342205; doi:10.1186/s13068-015-0201-7)
Supplement: Additional file 3: Table S1. — Genes related to c-di-GMP synthesis and hydrolysis in C. saccharolyticus and C. owensensis. [file 13068_2015_201_MOESM3_ESM.docx]

**Table S1 Genes related to c-di-GMP synthesis and hydrolysis in C. saccharolyticus and C. owensensis**

| **Gene Object ID** | **Locus Tag** | **Genome** | **Gene Product Name** |
| --- | --- | --- | --- |
| 640494395 | Csac_0020 | *C. saccharolyticus* | diguanylate cyclase/phosphodiesterase with PAS/PAC and GAF sensor(s) |
| 640494581 | Csac_0219 | *C. saccharolyticus* | diguanylate cyclase/phosphodiesterase |
| 640494737 | Csac_0377 | *C. saccharolyticus* | putative diguanylate cyclase |
| 640495105 | Csac_0756 | *C. saccharolyticus* | diguanylate cyclase |
| 640495170 | Csac_0821 | *C. saccharolyticus* | diguanylate cyclase/phosphodiesterase with PAS/PAC sensor(s) |
| 640496390 | Csac_2058 | *C. saccharolyticus* | diguanylate cyclase |
| 640496727 | Csac_2384 | *C. saccharolyticus* | diguanylate cyclase |
| 640496729 | Csac_2386 | *C. saccharolyticus* | putative adenylate/guanylate cyclase |
| 649735385 | Calow_0008 | *C. owensensis OL* | diguanylate cyclase |
| 649735646 | Calow_0281 | *C. owensensis OL* | diguanylate cyclase/phosphodiesterase |
| 649735737 | Calow_0385 | *C. owensensis OL* | diguanylate cyclase |
| 649735800 | Calow_0449 | *C. owensensis OL* | diguanylate cyclase/phosphodiesterase with pas/pac sensor(s) |
| 649736626 | Calow_1290 | *C. owensensis OL* | diguanylate cyclase |
| 649736928 | Calow_1586 | *C. owensensis OL* | diguanylate cyclase with gaf sensor |
| 649737255 | Calow_1920 | *C. owensensis OL* | diguanylate cyclase/phosphodiesterase |
| 649737500 | Calow_2179 | *C. owensensis OL* | diguanylate cyclase/phosphodiesterase |
| 649737555 | Calow_2237 | *C. owensensis OL* | diguanylate cyclase/phosphodiesterase with pas/pac sensor(s) |
