# Supplementary material for: Biofilm formation by designed co-cultures of Caldicellulosiruptor species as a means to improve hydrogen productivity
Source: Biotechnol Biofuels. 2015 Feb 12;8:19. doi: 10.1186/s13068-015-0201-7 (PMC4342205; doi:10.1186/s13068-015-0201-7)
Supplement: Supplementary file 2 [file 13068_2015_201_MOESM1_ESM.docx]

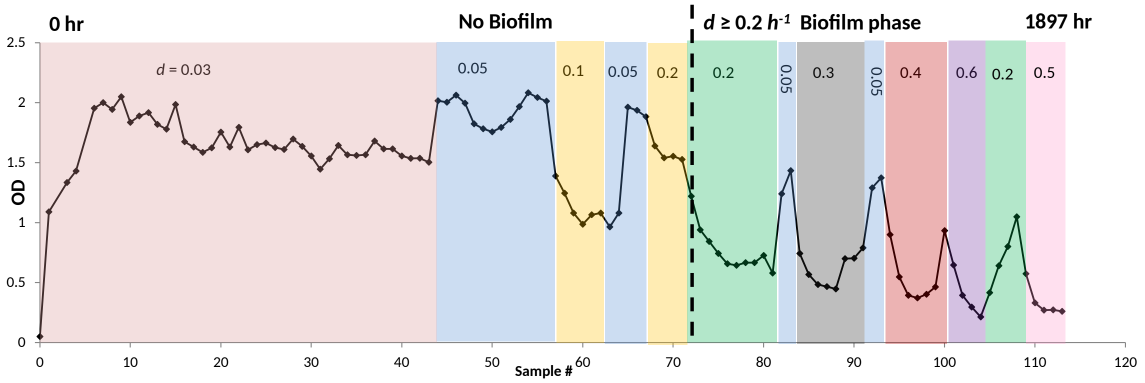

0 hr
No Biofilm
d ≥ 0.2 h-1 Biofilm phase
1897 hr
d = 0.03
0.05
0.1
0.05
0.2
0.2
0.05
0.3
0.05
0.4
0.6
0.2
0.5
OD
Sample #
